# Design and analysis of factorial clinical trials: The impact of one treatment's effectiveness on the statistical power and required sample size of the other

**DOI:** 10.1177/09622802231163332

**Published:** 2023-04-19

**Authors:** Stephen D. Walter, Ian J. Belo

**Affiliations:** 1 Department of Health Research Methods, Evidence, and Impact, 3710 McMaster University, Canada; 2 Department of Mathematics and Statistics, 3710 McMaster University, Canada

**Keywords:** Factorial studies, power, sample size, clinical trials, study design

## Abstract

Factorial allow for the simultaneous evaluation of more than one treatment, by randomizing patients to their possible combinations, including control. However, the statistical power of one treatment can be influenced by the effectiveness of the other, a matter that has not been widely recognized. In this paper, we evaluate the relationship between the observed effectiveness of one treatment and the implied power for a second treatment in the same trial, under a range of conditions. We provide analytic and numerical solutions for a binary outcome, under the additive, multiplicative, and odds ratio scales for treatment interaction. We demonstrate how the minimum required sample size for a trial depends on the two treatment effects. Relevant factors include the event rate in the control group, sample size, treatment effect sizes, and Type-I error rate thresholds. We show that that power for one treatment decreases as a function of the observed effectiveness of the other treatment if there is no multiplicative interaction. A similar pattern is observed with the odds ratio scale at low control rates, but at high control rates, power may increase if the first treatment is moderately more effective than its planned value. When treatments do not interact additively, power may either increase or decrease, depending on the control event rate. We also determine where the maximum power occurs for the second treatment. We illustrate these ideas with data from two actual factorial trials. These results can benefit investigators in planning the analysis of factorial clinical trials, in particular, to alert them to the potential for losses in power when one observed treatment effect differs from its originally postulated value. Updating the power calculation and modifying the associated required sample size can then ensure sufficient power for both treatments.

## Background

In this paper, we examine how the statistical power for one treatment effect in a binary-outcome 2 × 2 factorial randomized controlled trial (fRCT) may be influenced by the effectiveness of the other treatment. fRCTs are a widely used class of clinical trial designs that permit the evaluation of two or more treatments (or more generally, factors) simultaneously. These designs are more efficient in requiring fewer total participants compared to conducting two separate, single-factor trials.

fRCTs can be used to evaluate the effectiveness of multiple treatments by randomly assigning participants to subgroups who receive experimental treatments either alone or in combination or appropriate controls. The simplest such trial design involves two treatments (A and B), where participants receive either A alone, B alone, both A and B, or neither treatment. As in single-factor trials, the controls associated with each treatment receive either a placebo or an alternative treatment regimen (such as standard care). In combination, there are then four treatment subgroups (two treatments each at two levels). For clarity, we will refer to the patients receiving neither A nor B as the control group.

In planning an fRCT, investigators typically determine the minimum number of patients required by using postulated effect sizes for each treatment, along with a specified level of power. In certain situations, the effect of one treatment may be analysed before the second treatment is evaluated. This can occur at the time of a formal interim analysis, or more generally where the analysis of one treatment is seen by the investigators as a priority over the other, or indeed in any situation where the two treatments are not analysed simultaneously. If the first treatment effect is found to differ from its value proposed originally at the stage of study design and protocol creation, there are then consequences for the future analysis of the second treatment. Specifically, the originally projected marginal effect for the second treatment and its associated power is no longer valid, and should be updated. Depending on the direction and magnitude of the first treatment effect, there could be an increase or decrease in power for the second treatment, compared to the protocol estimate. Although increases in power for the second treatment would clearly benefit investigators, scenarios where power decreases occur should necessitate a larger sample to compensate.

In binary outcome fRCTs in particular, this issue is further complicated because the anticipated event rate in the patient subgroup receiving both treatments – and by extension their associated marginal effects – depends on the scale of measurement chosen to define treatment interactions. Separate examinations of the scenarios in which we assume that there is no interaction on each scale (additive, multiplicative or odds ratio) are therefore needed.

We will limit our attention to trials with a binary outcome, whose response rates in the 2 × 2 design are denoted by $p_{11}$ (patients receiving both active treatments), $p_{10}$ (active treatment A and control for B), $p_{01}$ (active treatment B and control for A) and $p_{00}$ (controls for both treatments). These trials can examine the individual effects of each treatment as well as potential treatment interactions, the latter case being where the effectiveness of one treatment depends on the level of the other treatment. However, if the treatments act independently (i.e., there is no interaction), fRCTs have an additional advantage of efficiency, meaning that factorial trials require fewer total participants than would be needed to conduct two separate trials with only a single treatment used in each one. This advantage emerges because fRCTs use the information from all their participants in the analysis of both treatments. The effects of both treatments can then be considered simultaneously in just one sample of patients. Empirically, the factorial trial design is most commonly conducted because of this advantage: a review by McAlister et al.^1^ found that among 44 binary outcome 2 × 2 fRCTs, 82% were conducted for the main purpose of efficiency, with the remaining 18% conducted mainly to assess interactions.

In single-factor trials, the treatment effect size is estimated by comparing the response rates in the experimental treatment and control groups. However, in a factorial trial, it is the marginal response rates that are used to make this comparison. Thus, outcomes from all patients are included in the analyses of both treatments, with the same sample size as would have been needed to analyse them separately. The marginal comparison can be misleading if an interaction exists, and even small interactions can lead to substantial bias.^1^ Since the presence or magnitude of interactions are rarely known in advance, preliminary interaction testing is sometimes used to decide if they can be ignored. Nevertheless, fRCTs are frequently underpowered to detect interactions, and some investigators use inappropriate methods to assess them.^2,3^ Moreover, the definition of interaction depends on which scale of measurement is being used. In particular, the absence of interaction on one scale directly implies interaction on a different scale.^4–6^ In this paper, we focus on trials where we assume in turn that there is no interaction on each given measurement scale (additive, multiplicative or odds ratio), and where the analysis of the marginal effects is therefore valid.

The number of participants needed to achieve a desired level of power for each treatment effect in a fRCT may be determined using standard methods. Sample sizes are typically calculated for the postulated effects of each treatment separately (assuming no interaction), with the larger size being taken as the requirement for the trial. This practice ensures sufficient power for both treatments. If the sample size required for one treatment is larger than for the other, the resultant total sample size for the trial will provide additional power for the treatment with the smaller required sample size.

It is critical to note that the marginal event rates anticipated for each treatment will necessarily depend on the magnitude of the other treatment effect, even without interactions. If the effect of A is analysed first and is found to differ from its originally planned value, investigators will presumably now use the updated empirical estimate as the best available information on the effect of A. The expected marginal event rates for B then will be altered, and by association, its power. This is true even if investigators maintain their original postulated value for the effect of B. If, based on the analysis of A, investigators realize that the power for B has changed, they will be better positioned to adapt the study appropriately. For example, if the power has gone down, they may recruit additional patients to compensate. However, this decision is complicated by the dependence of interaction on the measurement scale. In particular, the expected event rate in the combined treatment group (A ^+ ^B^+^) depends not only on both the treatment main effects but also on the assumed scale for the joint treatment effect.

The main focus of this paper is to elucidate how the power to detect one treatment effect in a fRCT is impacted by the effect of the other treatment. We consider scenarios where the treatments are assumed to have no interaction on either the additive, multiplicative, or odds ratio scales. We develop relevant formulae for power and associated required sample sizes. We then examine the expected changes in power and required sample sizes for one treatment, once we have obtained a treatment effect estimate for the other treatment in a previous analysis; we do this separately for each measurement scale. Numerical and graphical works show how the power for one treatment changes as a function of the effectiveness of the other, in a variety of scenarios. We also demonstrate the sample size adjustment that is needed to maintain the desired level of power in the second treatment analysis. We use two real-world examples of fRCTs to illustrate these ideas. Finally, we provide general recommendations on how to apply our findings to future trials.

## Methods

When planning a single treatment clinical trial, investigators typically postulate values of treatment effectiveness in order to determine the statistical power of the study. If the outcome variable is binary, the estimated power to detect the difference between the randomized treatment and control arms is often calculated using a normal approximation to the two underlying binomial distributions. The power to detect the difference between two independent response rates $p_{1}$ and $p_{2}$ , using a two-sided test with equal sample sizes *n* per group is given by

(1) $$\begin{array}{r} \text{Power}=\Phi \left[\frac{|p_{2}-p_{1}|\sqrt{n}-Z_{\left(1-\alpha /2\right)}\sqrt{2\bar{p}(1-\bar{p})}}{\sqrt{p_{1}(1-p_{1})+p_{2}(1-p_{2})}}\right] \end{array}$$

where $\bar{p}=(p_{1}+p_{2})/2$ , Φ(.) is the standard normal density function and $Z_{\left(1-\alpha /2\right)}$ is the *Z*-score associated with the $100(1-\alpha /2{)}^{\text{th}}$ percentile of the standard normal distribution. For a one-sided test, the $Z_{\left(1-\alpha /2\right)}$ term is replaced by $Z_{\left(1-\alpha \right)}$ , but we will focus our attention on two-sided testing. As noted earlier, power is usually estimated for each treatment separately in a factorial trial, using marginal values for the postulated experimental and group response rates. These marginal estimates for treatment are obtained by averaging the response rates for the treatment-present and treatment-absent groups across the levels of the other treatment.

Equation (1) can be inverted to determine the minimum sample size n* required to yield a specified power, given a postulated treatment effect, given by:

(2) $$\begin{array}{r} n^{*}={\left(\left[\sqrt{2\bar{p}(1-\bar{p})}\times Z_{\left(1-\propto /2\right)}-\sqrt{p_{1}(1-p_{1})+p_{2}(1-p_{2})}\times Z_{\left(1-\beta \right)}\right]/|p_{2}-p_{1}|\right)}^{2} \end{array}$$

where β is the Type II error rate. A target power of 0.80 (with β = 1–0.80 = 0.20) is commonly suggested by clinical guidelines.^7^ A more accurate, continuity-corrected estimate is^8^ given by:

(3) $$\begin{array}{r} n=\frac{n^{*}}{4}{\left(1+\sqrt{1+\frac{4}{n^{*}|p_{2}-p_{1}|}}\right)}^{2} \end{array}$$

we use (3) to obtain the required sample size for each treatment effect, adopting $p_{1}$ and $p_{2}$ as the anticipated marginal response rates for each treatment. This leads to two sample sizes, and their maximum is taken as the total sample size for the trial. This yields the target power for the treatment with the larger estimated *n*, and additional power for the treatment with the smaller *n*.

### Scales of measurement

On the additive scale, effect sizes are defined in terms of absolute risk reduction, or the arithmetic difference in response rates between patients receiving an experimental treatment or control. On this scale, the interaction is defined as:

$$p_{11}-p_{10}-p_{01}+p_{00}$$

where $p_{11}$ is the response rate for patients receiving both treatments A and B, $p_{10}$ is the response rate for patients receiving A and the control for B, $p_{01}$ is the response rate for patients receiving B and the control for A, and $p_{00}$ is the response rate for patients receiving controls for both A and B. If this quantity is zero, there is no additive interaction, while positive or negative values indicate positive or negative interactions, respectively.

On the multiplicative scale, the event rates in the three active treatment groups and the double control group give risk ratios: $RR_{\left(11\right)}=p_{11}/p_{00}$ , $RR_{\left(10\right)}=p_{10}/p_{00}$ , and $RR_{\left(01\right)}=p_{01}/p_{00}$ . The interaction is then defined by:

$$\frac{RR_{\left(11\right)}}{RR_{\left(10\right)}\times RR_{\left(01\right)}}=\frac{p_{11}\times p_{00}}{p_{10}\times p_{01}}$$

If this ratio equals 1, there is said to be no interaction, while ratios greater or less than 1 indicate positive or negative interactions, respectively.

The odds ratios for the three experimental treatment groups are $\text{O}{\text{R}}_{\left(11\right)}=\frac{p_{11}/(1-p_{11})}{p_{00}/(1-p_{00})}$ , $\text{O}{\text{R}}_{\left(10\right)}=\frac{p_{10}/(1-p_{10})}{p_{00}/(1-p_{00})}$ , and $\text{O}{\text{R}}_{\left(01\right)}=\frac{p_{01}/(1-p_{01})}{p_{00}/(1-p_{00})}$ . Interaction on this scale is defined by

$$\frac{\text{O}{\text{R}}_{\left(11\right)}}{\text{O}{\text{R}}_{\left(10\right)}\times \;\text{O}{\text{R}}_{\left(01\right)}}$$

which takes the value 1 with no interaction, and values greater than 1 or less than 1 correspond to positive or negative interactions.

### Relationship between power and scales of measurement

To generate sample size estimates in a factorial trial, investigators must postulate both the simple and combined effects of each treatment. In particular, the anticipated outcome in the combined treatment group depends on the scale of measurement used to define treatment effects. This in turn influences the anticipated marginal response rates for both treatments, and the corresponding sample size required to detect them at a specified level of power. We now show analytically how the anticipated power and required sample size for one treatment may change as a consequence of the other treatment having an effect that is different from its planned value. We do this separately for each measurement scale, assuming no interaction in each case, and with equal sample sizes in each of the four treatment arms.

With the additive scale, we denote $\Delta_{A}=p_{10}-p_{00}$ and $\Delta_{B}=p_{01}-p_{00}$ as the postulated effects of treatments A and B, respectively. The expected marginal response rates in the control (B absent) and treatment (B present) groups used to assess the effect of B are, assuming no additive interaction, respectively:

$$p_{1}=\frac{p_{00}+(p_{00}+\Delta_{A})}{2}=p_{00}+\frac{\Delta_{A}}{2}$$

$$p_{2}=\frac{\left(p_{00}+\Delta_{B})+(p_{00}+\Delta_{A}+\Delta_{B}\right)}{2}=p_{00}+\Delta_{B}+\frac{\Delta_{A}}{2}$$

Suppose A is analysed first, and found to have an effect that is different from its protocol value. Based on this observation, the investigators should then update their value $\Delta_{A}$ to $\left(\Delta_{A}+k\right)$ , where *k* is bounded $\left(-(p_{00}+\Delta_{A})\leq k\leq 1-p_{00}-\Delta_{A}\right)$ to avoid impossible rates outside the interval (0,1). The updated marginal response rates for *B* are now:

(4) $$\begin{array}{r} p_{1}^{\prime }=p_{00}+\frac{\Delta_{A}+k}{2}\;\text{and}\;p_{2}^{\prime }=p_{00}+\Delta_{B}+\frac{\Delta_{A}+k}{2} \end{array}$$

and these values can be used in (1) to update the power for *B*, and to calculate the corresponding change in power. We can similarly calculate the change in sample size needed to retain a given target level of power for *B*. If the revised sample size for *B* is ${\text{n}}_{{\text{B}}^{\prime }}$ , then the implied change in the total required sample size of the trial is $\Delta n=n_{B^{\prime }}-\text{max}(n_{A},n_{B})$ .

Next, for the multiplicative case, we let $\theta_{\text{A}}=p_{10}/p_{00}$ and $\theta_{\text{B}}=p_{01}/p_{00}$ denote the postulated effects of treatments A and B. The expected marginal rates with B absent or present respectively, and representing the effect of B are now:

$$p_{1}=\frac{p_{00}+(p_{00}\theta_{\text{A}})}{2}=\frac{p_{00}(1+\theta_{\text{A}})}{2}$$

$$p_{2}=\frac{p_{00}\theta_{\text{B}}+(p_{00}\theta_{\text{A}}\theta_{\text{B}})}{2}=\frac{p_{00}\theta_{\text{B}}(1+\theta_{\text{A}})}{2}$$

when treatment A is analysed first, its observed effect is $k\theta_{\text{A}}$ for some k>0 is a factor indicating the discrepancy between the observed and postulated treatment effects for A. The new anticipated response rates for B become:

(5) $$\begin{array}{r} p_{1}^{\prime }=\frac{p_{00}(1+k\theta_{\text{A}})}{2}\;\text{and}\;p_{2}^{\prime }=\frac{p_{00}\theta_{\text{B}}(1+k\theta_{\text{A}})}{2} \end{array}$$

Similarly to before, we can now use (5) to assess the implied changes in power and sample size for treatment B.

Finally, for the odds ratio scale, we let $\psi_{\text{A}}$ and $\psi_{\text{B}}$ again represent the postulated effects of treatments A and B, respectively, on the odds scale of measurement. If there is no interaction, the expected marginal event rates for B are now:

$$p_{1}=\frac{p_{00}+(p_{00}\psi_{\text{A}})}{2}=\frac{p_{00}(1+\psi_{\text{A}})}{2}$$

$$p_{2}=\frac{\psi_{\text{A}}\psi_{\text{B}}p_{00}(1-p_{00})}{2[\left(1-\psi_{\text{A}}p_{00})(1-\psi_{\text{B}}p_{00})+\psi_{\text{A}}\psi_{\text{B}}p_{00}(1-p_{00}\right)]}+\frac{p_{00}\psi }{2}$$

If the analysis of treatment A now gives an observed effect of $k\psi_{\text{A}}$ for some k>0 , then the revised marginal proportions for treatment B become:

$$p_{1}^{\prime }=\frac{p_{00}+(p_{00}k\psi_{\text{A}})}{2}=\frac{p_{00}(1+k\psi_{A})}{2}$$

$$p_{2}^{\prime }=\frac{k\theta_{\text{A}}\psi_{\text{B}}p_{00}(1-p_{00})}{2[\left(1-k\psi_{\text{A}}p_{00})(1-\psi_{\text{B}}p_{00})+k\psi_{\text{A}}\psi_{\text{B}}p_{00}(1-p_{00}\right)]}+\frac{p_{00}\psi_{\text{B}}}{2}$$

which again indicates the implied changes to the power and required sample size for B.

### Power functions: Analytic approach

We will assume that both treatments are intended to reduce the outcome event rate (e.g., the death rate), and therefore we will consider scenarios in which the first treatment analyzed either reduces the event rate or has a null effect. We will examine cases where the second treatment reduces the event rate, but omit the trivial case where it has no effect (because this always corresponds to its power being α , regardless of the first treatment's effect). Of particular interest are the cases when the second treatment's power attains a maximum or minimum, as a function of the effect of the first treatment. We now attempt to determine these points analytically.

With the additive scale, given a postulated $\Delta_{A}$ , we need to determine the value of *k* such that the observed effect of treatment A, $\Delta_{\text{A}}+\text{k}$ , maximizes the power for treatment B. On this scale, the power for B is obtained from (1) with the marginal event rates given by (4): with this and the other measurement scales, in our analytic development, we assume that the sample size is large enough that the second term in the numerator of (1) is effectively ignorable, and so we use a simplified expression:

$$\text{Power}=\Phi \left[\frac{\left|p_{2}^{\prime }-p_{1}^{\prime }\right|}{\sqrt{[p_{1}^{\prime }(1-p_{1}^{\prime })+p_{2}^{\prime }(1-p_{2}^{\prime })]/n}}\right]=\Phi (Q)$$

However, in our numerical evaluations (described later), we will retain the full power expression. To examine the extrema of power for treatment B, we require

(6) $$\begin{array}{r} \frac{d(\text{Power})}{df}=(\phi (Q))\left(\frac{dQ}{df}\right) \end{array}$$

where $\phi (Q)=\frac{1}{\sqrt{2\pi }}exp\left(\frac{-Q^{2}}{2}\right)$ is the probability density function for the standard normal distribution, and $f=\Delta_{\text{A}}+k$ . Differentiating by parts, after simplification we have:

$$\frac{dQ}{df}=\frac{-\sqrt{n}(1-2p_{00}-f-\Delta_{B})(\left|p_{2}^{\prime }-p_{1}^{\prime }\right|)}{2{\left[\left(p_{00}+\text{f}/2)(1-(p_{00}+\text{f}/2))+(p_{00}+\Delta_{B}+\text{f}/2)(1-(p_{00}+\Delta_{B}+\text{f}/2)\right)\right]}^{\frac{3}{2}}}$$

We note that ϕ(Q)→0 as Q→±∞. This occurs in the extreme tails of the distribution of *Q*, or when $(p_{1}^{\prime }(1-p_{1}^{\prime })+p_{2}^{\prime }(1-p_{2}^{\prime }))/n\to 0$ , and this is possible with several solutions. First, we may have $p_{1}^{\prime }=p_{2}^{\prime }=0,$ or $p_{1}^{\prime }=p_{2}^{\prime }=1,$ which are degenerate cases in which the event rates are always 0 or 1; in both these cases, there is also no effect of either treatment. Another solution is $f=1-\Delta_{B}\pm \sqrt{1-{\Delta_{B}}^{2}}-2p_{00}$ , which can occur when f=0 , $\Delta_{B}=0$ , and $p_{00}=1$ ; this is also a scenario where the event rates are always 1.

Turning attention to the second component of (6), we note that $\frac{dQ}{df}=0$ if either $|p_{2}^{\prime }-p_{1}^{\prime }|=0$ or $(1-2p_{00}-f-\Delta_{B})=0$ . The former occurs when neither treatment has an effect, and therefore the power of B does not change after the analysis of A; the latter provides a non-trivial solution, and its numerical evaluations are discussed later.

On the multiplicative scale, we again need to determine the value of *k* which maximizes the power of treatment B. The power is calculated from (1) (again ignoring the second numerator term) with marginal event rates now given by (5). Analogously to the additive case, we calculate the partial derivative of power (cf. equation 6) with respect to $g=(1+k\theta_{\text{A}})$ , and after simplification we obtain:

$$\frac{dQ}{dg}=\frac{\sqrt{n}(p_{00}-{p_{00}}^{2}g+p_{00}\theta_{B}-{p_{00}}^{2}g{\theta_{B}}^{2})[\left|p_{2}^{\prime }-p_{1}^{\prime }\right|]}{4{\left(\left(\frac{p_{00}g}{2}\right)\left(1-\frac{p_{00}g}{2}\right)+\left(\frac{p_{00}g\theta_{B}}{2}\right)\left(1-\frac{p_{00}g\theta_{B}}{2}\right)\right)}^{3/2}}+\frac{\left(\frac{p_{00}(1-\theta_{B})}{2}\right)}{\sqrt{p_{1}^{\prime }(1-p_{1}^{\prime })+p_{2}^{\prime }(1-p_{2}^{\prime })/n}}$$

Here, ϕ(Q)=0 when $p_{1}^{\prime }=p_{2}^{\prime }=0$ or $p_{1}^{\prime }=p_{2}^{\prime }=1$ , and the only other solution to $(p_{1}^{\prime }(1-p_{1}^{\prime })+p_{2}^{\prime }(1-p_{2}^{\prime }))=0$ (which implies ϕ(Q)=0 ) is $k\theta_{\text{A}}=\frac{4-(p_{00}(1+\theta_{B}))}{p_{00}(1+\theta_{B})}$ . Since $0\leq k\theta_{\text{A}}\leq 1$ , this solution is minimized at $k\theta_{\text{A}}=1$ when $p_{00}$ and $\theta_{B}$ are maximized, i.e., when $p_{00}=\theta_{\text{B}}=1$ ; this is a degenerate case when all event rates are 1. The second term of $\frac{dQ}{dg}$ is strictly negative at all constrained values of the parameters, and has no real roots apart from when $p_{1}^{\prime }=p_{2}^{\prime }$ (when neither treatment has any effect). Together, this means that except for the degenerate cases, power is a strictly negative function of *f*, meaning that the power for B always decreases as the strength of the A effect increases, with maximum power occurring when A has no effect. As stated at the beginning of this section, we assume that both treatments either reduce the event rate or have a null effect, meaning that this maximum exists under the condition where $0\leq k\theta_{\text{A}}\leq 1$ .

For the odds ratio scale, if we denote the observed effect of A by *c =* $k\text{O}{\text{R}}_{\text{A}}$ , the updated marginal event rates for B are

$$p_{1}^{\prime }=\frac{p_{00}(1-p_{00})}{2(1-p_{00}+c)}\;p_{2}^{\prime }=\frac{p_{00}\text{O}{\text{R}}_{B}(2c\text{O}{\text{R}}_{B}p_{00}-cp_{00}-p_{00}+c+1)}{2(\text{O}{\text{R}}_{B}p_{00}-p_{00}+1)(c\text{O}{\text{R}}_{B}p_{00}-p_{00}+1)}$$

With this scale, we have:

$$\frac{dQ}{dc}=\frac{d}{dc}\left[\frac{1}{\sqrt{p_{1}^{\prime }(1-p_{1}^{\prime })+p_{2}^{\prime }(1-p_{2}^{\prime })/n}}\right][\left|p_{2}^{\prime }-p_{1}^{\prime }\right|]+\frac{d}{dc}[\left|p_{2}^{\prime }-p_{1}^{\prime }\right|]\left[\frac{1}{\sqrt{p_{1}^{\prime }(1-p_{1}^{\prime })+p_{2}^{\prime }(1-p_{2}^{\prime })/n}}\right]$$

and it can be shown that:

$$\frac{d}{dc}[\left|p_{2}^{\prime }-p_{1}^{\prime }\right|]=\frac{p_{00}\left(1+\text{O}{{\text{R}}_{\text{B}}}^{2}\frac{c{p_{00}}^{2}}{{\left(1-p_{00}\right)}^{2}}-\text{O}{\text{R}}_{\text{B}}(1+c^{2}+1)\right)}{2{\left(1+\frac{cp}{1-p}\right)}^{2}{\left(1+\frac{\text{O}{\text{R}}_{\text{B}}cp}{1-p})\right)}^{2}}$$

Also, after denoting $m=\frac{p_{00}}{1-p_{00}}$ ,

$$\frac{d}{dc}\left[\frac{1}{\sqrt{p_{1}^{\prime }(1-p_{1}^{\prime })+p_{2}^{\prime }(1-p_{2}^{\prime })/n}}\right]$$

$$\begin{array}{r} ={\left[\left(\frac{-p_{00}}{2}-\frac{cm}{2(cm+1)}\right)\left(\frac{p_{00}}{2}+\frac{cm}{2(cm+1)}-1\right)+\left(\frac{-\text{O}{\text{R}}_{\text{B}}}{2(\text{O}{\text{R}}_{\text{B}}m+1)}-\frac{\text{O}{\text{R}}_{\text{B}}cm}{2(\text{O}{\text{R}}_{\text{B}}cm+1)}\right)\left(\frac{1}{n}\right)\right]}^{-1/2}\\ \times \left[\left(\frac{1}{n}\right)\left(\frac{-m^{2}p_{00}c-mp_{00}+m}{2{\left(cm+1\right)}^{3}}-\frac{\text{O}{\text{R}}_{\text{B}}m(\text{O}{\text{R}}_{\text{B}}-\text{O}{\text{R}}_{\text{B}}m+\text{O}{{\text{R}}_{\text{B}}}^{2}cm-1)}{2(\text{O}{\text{R}}_{\text{B}}m+1){\left(\text{O}{\text{R}}_{\text{B}}cm+1\right)}^{3}}\right)\right] \end{array}$$

The two portions above can then be combined into the full derivative $\frac{d(Q)}{dc}.$

As with the previous two scales, ϕ(Q)=0 when $p_{1}^{\prime }=p_{2}^{\prime }=0$ or $p_{1}^{\prime }=p_{2}^{\prime }=1$ . There are no other real solutions such that $p_{1}^{\prime }(1-p_{1}^{\prime })+p_{2}^{\prime }(1-p_{2}^{\prime })=0$ . For the second part of the derivative, the roots of $\frac{dQ}{dc}=0$ do not appear to have a closed-form solution, apart from the degenerate $p_{1}^{\prime }=p_{2}^{\prime }$ . We could not find a real solution to $\frac{d}{dc}\left[\frac{1}{\sqrt{p_{1}^{\prime }(1-p_{1}^{\prime })+p_{2}^{\prime }(1-p_{2}^{\prime })/n}}\right]=0$ , but we did identify that $\frac{d}{dc}[\left|p_{2}^{\prime }-p_{1}^{\prime }\right|]=0$ in two degenerate cases: $p_{00}=0$ (no events in the double-control group), and $\text{O}{\text{R}}_{\text{B}}=1$ (treatment B has no effect). A non-degenerate solution occurs when $c=k\text{O}{\text{R}}_{\text{A}}=\frac{\left(1-p_{00}\right)}{p_{00}\sqrt{\text{O}{\text{R}}_{\text{B}}}}$ . Under this condition, $\frac{dQ}{dh}=\frac{d}{dc}\left[\frac{1}{\sqrt{p_{1}^{\prime }(1-p_{1}^{\prime })+p_{2}^{\prime }(1-p_{2}^{\prime })/n}}\right][\left|p_{2}^{\prime }-p_{1}^{\prime }\right|]$ , and solving this for the effect of treatment A should correspond to the maximum power for treatment B. Our numerical evaluations bear this out.

## Results

### Power functions: Numerical evaluations

We now present numerical and graphical evaluations to examine how power varies in various trial scenarios. We limit our evaluations here to two-sided testing with α = 0.05. Manipulating the α-level or changing to one-sided testing modifies the specific values of power, but the general trends remain unaltered.

We assume that each treatment has a postulated effect size for the binary outcome, as described in the original trial protocol. Treatment A is analysed first and its observed effect size then replaces the original value. Trials are otherwise characterized by the control event rate for the outcome variable, and the threshold type I error rate (α) for significance testing. The evaluations of power require the sample size as input, and *vice versa.* In all cases, the four treatment subgroups are assumed to have equal sizes.

We selected a range of treatment effect sizes that reflect the design of most clinical trials in practice. With a binary outcome, one possible representation of a treatment effect is the absolute difference between two response rates, $\left|p_{1}-p_{2}\right|$ . One popular way to describe effect sizes in general was proposed by Cohen,^9^ who applies an approximately normalizing transformation ( γ) to *p*, and then calculates the difference (denoted by Cohen as *h*) in the scale of γ rather than *p*. This provides a more helpful indicator of the detectability of effect sizes, because equal differences in γ correspond to effect sizes that are approximately equally detectable. The relevant equations are

$$\begin{array}{r} \gamma =2\sin^{-1}\sqrt{p} \end{array}$$

$$\begin{array}{r} h=|\gamma_{1}-\gamma_{2}| \end{array}$$

Cohen also proposed guidelines to interpret the magnitude of the effect size for a given value of *h*, along with a range for the corresponding effect sizes in terms of $\left|p_{1}-p_{2}\right|$ , as follows:

1. Small effect size: h=0.2 ( $0.05\leq |p_{1}-p_{2}|\leq 0.10)$
2. Medium effect size: $h=0.5\;(0.20\leq |p_{1}-p_{2}|\leq 0.25)$
3. Large effect size: $h=0.8\;(0.35\leq |p_{1}-p_{2}|\leq 0.39)$

Note that not all possible additive differences are explicitly covered by these ranges. Also, these range descriptions are designed as an aid to a broad interpretation of study results, and they should not be recommended over more precise considerations pertinent to a specific study. However, they should be flexible enough to apply to alternative scales of measurement and control event rates. Note also that Cohen's defined effect sizes can be adopted for a variety of effect measures, including the risk ratio and odds ratio, as we discuss below. We adopted Cohen's definitions to determine the values of the event rates that then specify the scenarios that we evaluate, given the starting point of the control event rate.

For our evaluations that assumed no additive interaction, absolute additive rate differences from 0 to 0.25 were used for both treatments, which approximately covers Cohen's small and medium effect sizes. Large effects – corresponding to additive differences of about 0.35 or more – were excluded because they would often result in predicting negative response rates in the combined treatment group for small or medium control event rates. For example, if both treatments have additive effects of 0.35, the expected response rate in the combined treatment group would be negative for any control event rate less than 0.7.

With the risk ratio or odds ratio scales, relative risk effects from 1 (no effect) to 0.5 (50% relative risk reduction) were used for both treatments. This corresponds to odds ratio effects between zero and one depending on the base rate. Here, there is no possibility of predicting a negative response rate in the combined treatment group, so we used effect sizes up to Cohen's ‘large’ definition, starting with control event rates up to 0.8. A maximum of h≈0.85 is attained if a treatment reduces the event rate from 0.8 to 0.4 (relative risk reduction of 0.5, or equivalent OR of 0.167).

#### Additive scale

**Figure 1.**
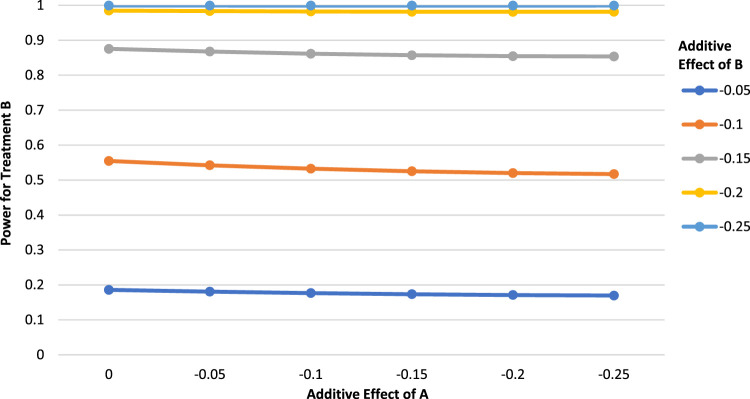
Power for treatment B as a function of the additive effect of treatment A ( $P_{00}=0.7,$ N=400 , α=0.05 , two-tailed test).

Figure 1 shows the power for B as a function of the effect of treatment A, with a relatively high control event rate of 0.70. We see that the power for B decreases slowly as a function of the effect of A, and the changes are small. Since the changes in power for B are relatively minor regardless of the effect of A, this implies that the power for B would not change greatly in settings where the observed effect of A is found to differ from its postulated effect in the first analysis. Consequently, there is no strong incentive for investigators to compensate with an increased sample size to maintain power. This is true in cases where the control event rate is high, as in the current example.

We showed previously that $k=1-2p_{00}-\Delta_{A}-\Delta_{B}$ is a solution to the point of zero-change in the power for treatment B. Using Figure 1 as an example, we can examine the case where $\Delta_{A}=-0.2$ and $\Delta_{B}=-0.15$ . Given a control event rate of $p_{00}=0.7,$ we should expect maximal or minimal power when k=1−2(0.7)+0.2+0.15=−0.05 . As an example, this would occur if the postulated effect of A is $\Delta_{A}=-0.2$ and an effect $\Delta_{A}+k=-0.25$ is actually observed. We found that the power for treatment B when $\Delta_{A}=-0.2$ and $\Delta_{B}=-0.15$ is 0.854. When $\Delta_{A}=-0.25$ , the power for treatment B remains at 0.854, in accordance with our algebraic result.

**Figure 2.**
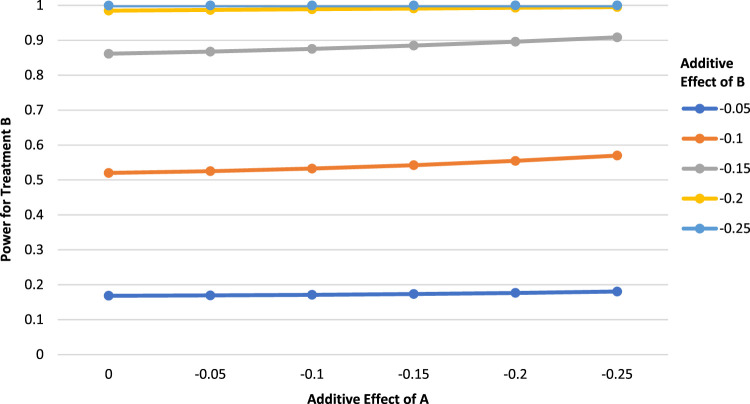
Power for treatment B as a function of the additive effect of treatment A ( $P_{00}=0.5,$ N=400 , α=0.05 , two-tailed test).

**Figure A1.**
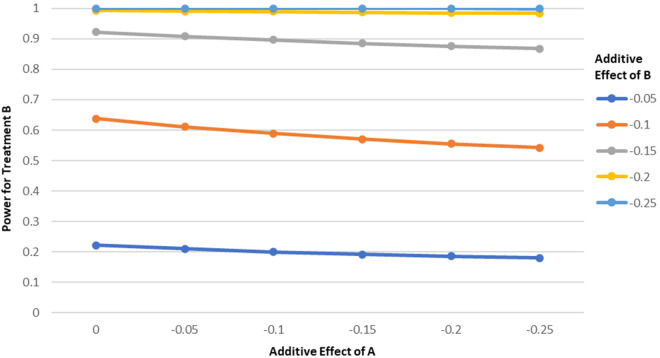
Power for treatment B as a function of the effectiveness of treatment A ( $P_{00}=0.8,$ N=400 , α=0.05 , two-sided test).

**Figure A2.**
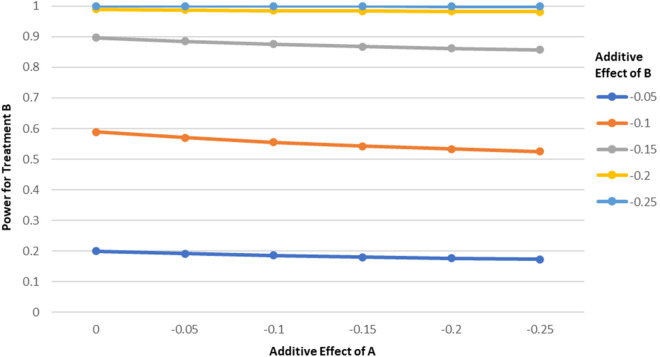
Power for treatment B as a function of the effectiveness of treatment A ( $P_{00}=0.75,$ N=400 , α=0.05 , two-sided test).

**Figure A3.**
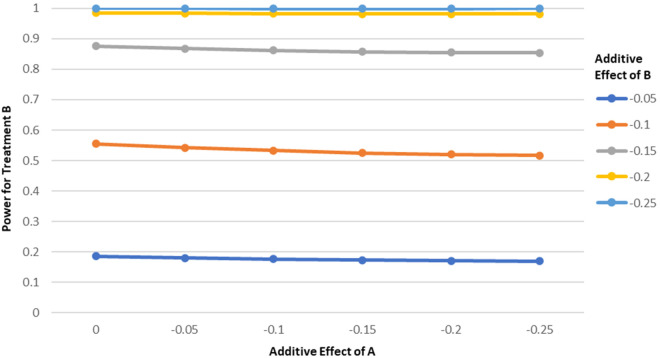
Power for treatment B as a function of the effectiveness of treatment A ( $P_{00}=0.7,$ N=400 , α=0.05 , two-sided test).

**Figure A4.**
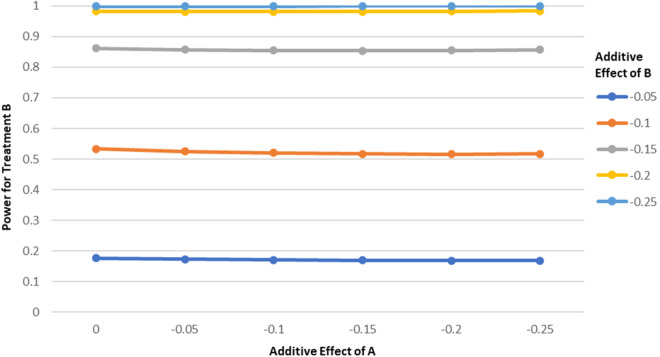
Power for treatment B as a function of the effectiveness of treatment A ( $P_{00}=0.65,$ N=400 , α=0.05 , two-sided test).

**Figure A5.**
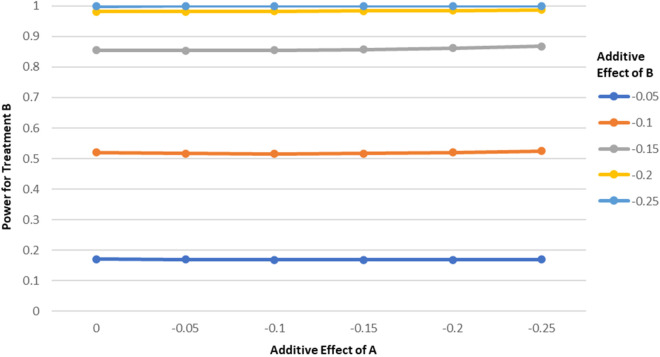
Power for treatment B as a function of the effectiveness of treatment A ( $P_{00}=0.60,$ N=400 , α=0.05 , two-sided test).

**Figure A6.**
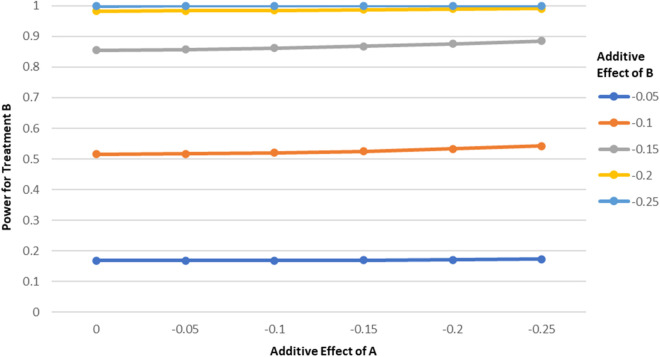
Power for treatment B as a function of the effectiveness of treatment A ( $P_{00}=0.55,$ N=400 , α=0.05 , two-sided test).

**Figure A7.**
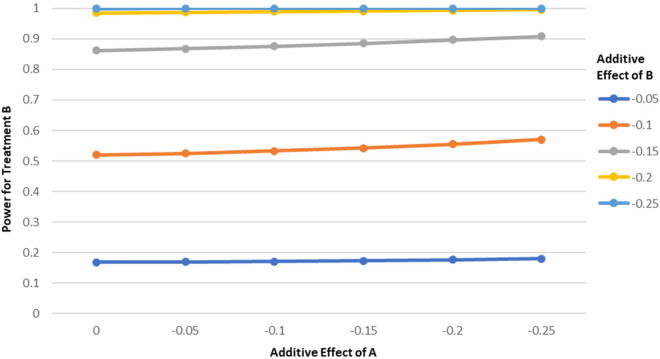
Power for Treatment B as a Function of the Effectiveness of Treatment A ( $P_{00}=0.50,$ N=400 , α=0.05 , Two-Sided Test).

In contrast, with moderate or low control event rates, an increased effect of A can result in a slightly increased power for B. As an example, Figure 2 is similar to before, except that the control event rate has been reduced to 0.5. We now see that a stronger effect of A is associated with increased power for B. For example, using Figure 2, suppose that a 2 × 2 fRCT is planned with a control rate of 0.5 and postulated additive effects $\Delta_{A}=-0.05,\Delta_{B}=-0.15,$ with 400 participants. In this scenario, treatment B has a power of approximately 0.867. However, if it is found that $\Delta_{A}=-0.1$ , corresponding to a stronger additive effect than postulated, the power for treatment B increases very slightly to approximately 0.875. In general, when holding the other parameters constant, starting with a high control event rate will result in small power decreases for B, which gradually reduce in magnitude and evolve to small power increases if the control event rate becomes lower. At which specific control event rate the power relationship shifts from decreases to increases depends on the settings of the other parameters, particularly the sample size, but is generally in the range of 0.5–0.6. Figure A1–A7 in the Appendix provide additional examples that show this gradual change in the power relationship while moving through a wide range of control event rates. Overall, while either power increases or decreases exist, they tend to be small.

#### Multiplicative scale

We now assume no interaction on the multiplicative scale, a control rate of 0.3, a total sample size of 2000 (500 per combined treatment subgroup), and α=0.05 (two-sided test). As an example, suppose a 2 × 2 fRCT is planned with postulated multiplicative effects of 0.8 for both treatments. This would yield a power of 0.804 for treatment B. Suppose treatment A is analysed first and is estimated to have no effect (i.e., a multiplicative effect of *k *= 1). Updating with this new information gives an estimated power for B of 0.856, about 6% more than it was at the study outset. This corresponds to a large relative decrease (28%, from 0.20 to 0.144) in the type II error when testing the effect of treatment B.

Alternatively, suppose that A has been found to be greatly more effective than anticipated originally, with an observed multiplicative effect of 0.5. Now, the updated power for B falls to 0.707, an absolute decrease of over 9%. This represents a 46.5% relative increase (from 0.20 to 0.293) in the type II error rate for the test of B. Cases such as this, with non-trivial reductions in power, may prompt investigators to adapt their study design, by recruiting more participants prior to the analysis of the second treatment B.

We continue with this example, by evaluating the number of additional patients needed to restore power to its original level; see Table 1 for a typical case, using (3) rounded up to the nearest integer. The minimum total sample in each case was determined by separately calculating the sample size required to detect each treatment effect with a power of 0.80, and then taking the maximum of the two for the trial sample size. Retaining the multiplicative effect of 0.80 for both treatments, and a control event rate of 0.30, a minimum sample size of 2054 patients would be needed to achieve 80% power. If the analysis for treatment A is conducted first and its multiplicative effect is found to be 0.50, the minimum required sample size needed to achieve a power of 0.80 for treatment B (still assuming that its effect is 0.8) is 2592, which is 528 patients more than the original target, or equivalently an approximately 26% increase in the study size. Note that cases in the first column of Table 1 would require an infinite sample size to achieve 80% power, as in this column the multiplicative effect of treatment A is 1 (has no effect). Table 2 shows the sample size required to maintain the desired power for treatment B in this case, for a variety of parameter settings.

**Table 1.** Minimum required total sample size as a function of the effectiveness of treatment A and treatment B, assuming no multiplicative interaction ( $P_{00}=0.3,$ Power=0.8 , α=0.05 , two-tailed test).

Total *N*		Multiplicative Effect of A										
Multiplicative effect of B		1	0.95	0.9	0.85	0.8	0.75	0.7	0.65	0.6	0.55	0.5
	**0**.**95**	#N/A	30196	31304	32472	33706	35012	36392	37856	39412	41068	42836
	**0**.**9**	#N/A	31304	7770	8058	8360	8680	9020	9378	9758	10166	10600
	**0**.**85**	#N/A	32472	8058	3552	3686	3824	3970	4128	4294	4470	4660
	**0**.**8**	#N/A	33706	8360	3686	2054	2130	2212	2300	2390	2488	2592
	**0**.**75**	#N/A	35012	8680	3824	2130	1350	1402	1454	1512	1574	1640
	**0**.**7**	#N/A	36392	9020	3970	2212	1402	962	998	1038	1080	1124
	**0**.**65**	#N/A	37856	9378	4128	2300	1454	998	724	754	784	816
	**0**.**6**	#N/A	39412	9758	4294	2390	1512	1038	754	570	592	614
	**0**.**55**	#N/A	41068	10166	4470	2488	1574	1080	784	592	460	480
	**0**.**5**	#N/A	42836	10600	4660	2592	1640	1124	816	614	480	382

**Table 2.** Minimum required sample size for treatment B as a function of the effectiveness of treatment A and treatment B, assuming no multiplicative interaction: ( $P_{00}=0.3,$ Power=0.8 , α=0.05 , two-tailed test).

Sample size for treatment B		Multiplicative effect of A										
Multiplicative effect of B		1	0.95	0.9	0.85	0.8	0.75	0.7	0.65	0.6	0.55	0.5
	**0**.**95**	29142	30196	31304	32472	33706	35012	36392	37856	39412	41068	42836
	**0**.**9**	7242	7500	7770	8058	8360	8680	9020	9378	9758	10166	10600
	**0**.**85**	3198	3310	3428	3552	3686	3824	3970	4128	4294	4470	4660
	**0**.**8**	1786	1846	1912	1980	2054	2130	2212	2300	2390	2488	2592
	**0**.**75**	1134	1172	1212	1256	1302	1350	1402	1454	1512	1574	1640
	**0**.**7**	778	806	834	864	894	928	962	998	1038	1080	1124
	**0**.**65**	566	588	606	628	650	674	700	724	754	784	816
	**0**.**6**	430	444	460	474	492	510	528	548	570	592	614
	**0**.**55**	336	346	360	370	384	398	412	426	444	460	480
	**0**.**5**	270	278	286	298	306	316	330	340	354	368	382

**Figure 3.**
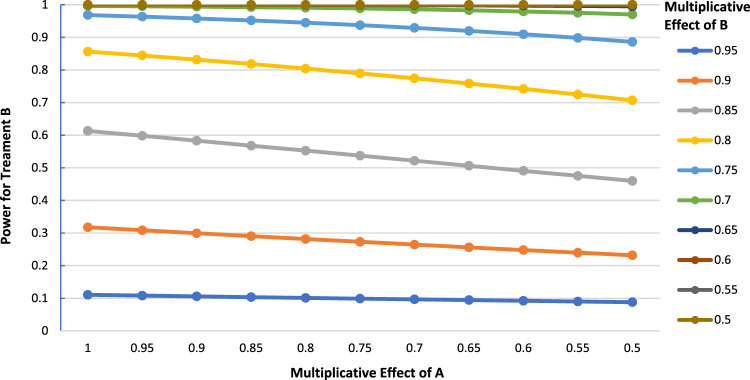
Power for treatment B as a function of the multiplicative effect of treatment A ( $P_{00}=0.3,$ N=2000 , α=0.05 , two-tailed test).

In general, greater effectiveness of A than anticipated always results in a decreased power for B when the treatments do not interact on the multiplicative scale, holding the other parameters constant. Broadly speaking, if treatment A's observed effect is within 10% of its postulated value, the impact it has on the power for B tends to be small (< 5%), regardless of the control event rate. Greater reductions in power are generally seen only when the observed effect of A is considerably larger than anticipated, in which case it would be beneficial to consider recruiting additional patients prior to the analysis of B. An interesting feature of the results is that the greatest reductions in power occur for intermediate values of the effect of treatment B. For instance, in Figure 3, the greatest power loss is when the multiplicative effect of B is 0.85 (power = 0.631 when the effect of A is 1 vs. power = 0.460 when the effect of A is 0.5, for a power loss of 0.153).

#### Odds ratio scale

**Figure 4.**
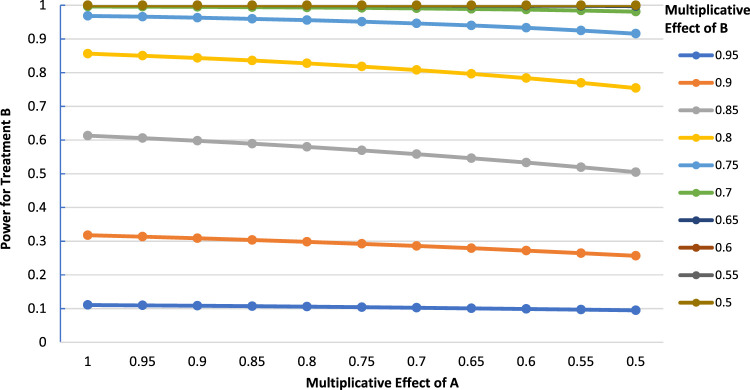
Power for treatment B as a function of the multiplicative effect of treatment A with no odds ratio interaction ( $P_{00}=0.3,$ N=2000 , α=0.05 , two-tailed test).

**Figure 5.**
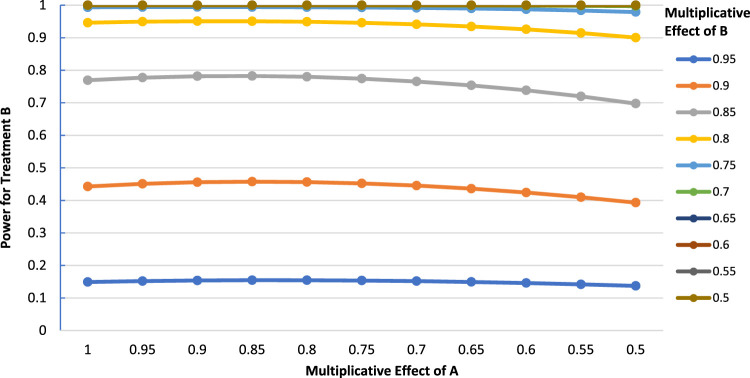
Power for treatment B as a function of the multiplicative effect of treatment A with no odds ratio interaction ( $P_{00}=0.7,$ N=600 , α=0.05 , two-tailed test).

Figure 4 shows corresponding results when there is no interaction on the odds ratio scale. The results are numerically close to those with the multiplicative scale, and the power curves exhibit the same general shape, particularly when the control event rate is low. In both cases, there is an overall decrease in the power for B as the effectiveness of A increases. However, there is a caveat as the control event rate becomes larger ( $P_{00}>0.6)$ . As the control event rate increases beyond this point, the odds ratio power curves now show slight concavity for some effect sizes of B. Figure 5 illustrates this effect.

Earlier, we posited that $k\text{O}{\text{R}}_{\text{A}}=\frac{\left(1-p_{00}\right)}{p_{00}\sqrt{\text{O}{\text{R}}_{\text{B}}}}$ should yield an approximate solution for the effect of A which maximizes the power for B. Using Figure 5 as an example, suppose we are interested in a situation when the postulated relative risk effect of treatment B is 0.85 (or equivalently OR = 0.63). Given that $P_{00}=0.7$ and treatment B reduces the event rate to (0.7)(0.85)=0.595 , we have that $\text{O}{\text{R}}_{\text{B}}=\frac{\left(0.595)/(1-0.595\right)}{\left(0.7)/(1-0.7\right)}=0.63$ . Then, we may calculate that the effect of treatment A to maximize the power for treatment B is $k\text{O}{\text{R}}_{\text{A}}=\frac{\left(1-0.7\right)}{0.7\sqrt{\left(0.63\right)}}=0.54$ ; this corresponds to A reducing the event rate from 0.7 to 0.56, or a relative risk effect of approximately 0.8 (equivalently, OR = 0.55) . Figure 5 indicates that the power for B is approximately 0.78 when the relative risk effect of A is 0.8. This is reasonably close to the true maximum power for B, which was verified by software to be 0.783, occurring when the multiplicative effect of treatment A is (more exactly) 0.863 (OR = 0.66).

In more generality, we find that slight power increases for B occur when the effect of A is small (from approximately 1 to 0.8), but as the effectiveness of A increases beyond this point, the power increases become smaller and then become decreases. Compared to the multiplicative case, the overall drop in power across the range of the effectiveness of A tends to be substantially smaller with a high control event rate. For example, the largest overall drop in power is seen when the effect of B is 0.85 (a difference of about 0.07 when the effect of A is 1 versus 0.5), but in the multiplicative case, the largest drop is also when the effect of B is 0.85, but there is then a larger power decrease (a difference of about 0.26 when the effect of A is 1 versus 0.5). Overall, the multiplicative and odds ratio evaluations show similar results when the control event rate is low, which is to be expected given the close numerical similarity between these indices in such cases. However, there is a clear divergence in the results between the two scales when the control event rate is high. The maximum power occurs at a moderate effect of the first treatment when the control event rate is high. At lower control event rates, the odds ratio power trends closely approximate those observed in the multiplicative case, with power for B decreasing as a function of the effectiveness of A.

### Illustrative examples

We now discuss two illustrative examples from actual clinical trials.

#### Example 1: Heyland et al.

In the factorial trial by Heyland et al.,^10^ critically ill patients experiencing multiorgan failure were randomly assigned to receive supplements of antioxidants, glutamine, both, or neither (placebo), with 28-day mortality taken as the primary outcome. The study was planned to detect a 25% relative risk reduction from 0.30 to 0.225 for both treatments (RR = 0.75, OR = 0.677), with a calculated total sample size of 1200, giving 80% power for each treatment. Two-tailed testing was used, with α=0.05 . The investigators were interested in assessing the main effects of each treatment, and they did not anticipate an interaction on the odds ratio scale. Assuming these conditions are met, the anticipated event rates for the trial are shown in Table 3.

**Table 3.** Postulated death rates (study by Heyland et al.).

		Antioxidants		
		Present	Absent	Total
Glutamine	Present	0.164	0.225	0.195
	Absent	0.225	0.300	0.263
	Total	0.195	0.263	

A total of 1223 patients were randomized to the four treatment groups, with 1218 patients being included in the primary analysis. Table 4 shows the observed results. After determining that there was no significant interaction between the two treatments on the odds ratio scale (*p* = 0.49), the investigators assessed the main effects of each treatment. There was no significant difference in mortality among patients with or without antioxidants (0.308 vs 0.288, OR = 1.09, p = 0.48), and a tendency for increased mortality among patients who received glutamine versus not (0.324 vs. 0.272, OR = 1.28, *p* = 0.05).

**Table 4.** Observed death rates (study by Heyland et al.).

		Antioxidants		
		Present	Absent	Total
Glutamine	Present	101/310 (0.326)	97/301 (0.322)	194/611 (0.318)
	Absent	89/307 (0.290)	76/300 (0.253)	165/607 (0.272)
	Total	190/617 (0.308)	173/601(0.288)	

We now explore the scenario in which the main effect of one of these treatments is analysed first and then we assess the resulting impact on the power for the other, again assuming no odds ratio interaction. Suppose the analysis for the main effect of antioxidants had been conducted first, with the investigators still blinded to the glutamine effect. The investigators had failed to find evidence of an antioxidants effect. If one updates their estimate of the antioxidant effect to zero based on this finding, this will subsequently change the anticipated marginal effect of glutamine and its associated power. Originally, a 25% risk reduction from 0.30 to 0.225 was anticipated for both interventions, implying anticipated marginal mortality rates of 0.195 and 0.263 for the glutamine-present versus glutamine-absent groups, respectively (Table 3). Once the main effect of antioxidants has been analyzed and found to have no effect if the investigators maintain their originally postulated value for the glutamine effect (and continue to assume no interaction), the anticipated event rates would be 0.225 and 0.300 for glutamine present or absent, respectively, regardless of their assignment to antioxidant or control, and hence the marginal event rates would be the same. If the analysis for glutamine is now carried out with the original target sample size of 1200, its power rises to approximately 0.84, representing a 20% relative decrease in the type II error rate of the test, thus reducing the chance of investigators failing to find a significant glutamine effect. This example thus illustrates the potential for power gains in the analysis for the second treatment.

#### Example 2: Poldermans et al.

Using data from the trial by Poldermans et al.,^11^ we demonstrate the potential for a power decrease. Patients at high risk of cardiac complications who were undergoing major vascular surgery were randomized to receive standard care with or without bisoprolol, a beta-blocker intended to reduce the risk of adverse cardiac events. The primary outcome was the proportion of patients who died of a cardiac event or had a non-fatal myocardial infarction. The study was designed to detect a 50% relative risk reduction (corresponding to OR = 0.41) in the primary outcome, from 0.30 to 0.15 at 80% power using a two-tailed test (α = 0.05). An interim analysis was conducted on the first 100 patients and a stop for benefit was planned if a significant difference (*p* = 0.001) in the primary endpoint was found between the bisoprolol and control groups. The interim results were 2/59 (3.4%, *n* = 59) in the bisoprolol group, and 18/53 (34.0%, *n* = 53) in the control (standard care) group (*p* < 0.001); this large effect led the investigators to stop the study at this point.

We now consider a scenario in which a hypothetical second treatment (denoted as B) had been administered in a factorial trial along with bisoprolol. Further suppose the investigators had planned for a 50% relative risk reduction in the primary outcome for both bisoprolol and B, with no interaction on the odds ratio scale. Under these assumptions, the anticipated event rates are shown in Table 5. Marginal event rates of 0.109 and 0.225 would be expected for the treatment-present and treatment-absent groups for B, respectively. To achieve 80% power for the effect of treatment B (two-tailed test, α = 0.05), a sample size of approximately 324 participants would be required.

**Table 5.** Postulated rates of non-fatal myocardial infarction or death (study by Poldermans et al.).

		Treatment B		
		Present	Absent	Total
Bisoprolol	Present	0.068	0.150	0.109
	Absent	0.150	0.300	0.225
	Total	0.109	0.225	

Next, suppose that the effect of bisoprolol is analysed first, and is observed to reduce the event rate of the primary outcome from 0.30 to 0.10 with treatment B absent, an effect that would be considered significant (*p* ≈ 0.02) but not enough to stop the study early for benefit. Analogously to the previous example, this new information on bisoprolol can be used to re-compute the power for the second factor, B. Updating the results while maintaining the assumption of no interaction, the anticipated results now become as in Table 6. The new marginal estimates for treatment B are 0.097 and 0.200 in the B-present and B-absent groups, respectively. If the analysis for B is carried out using the originally planned sample size of 324 participants, the power for the main effect of B falls to approximately 0.74, a relative 7.8% reduction from the original 80%, and a 30% relative increase in the type II error rate (from 0.20 to 0.26), meaning a greater chance of not finding a significant effect of B. To compensate, an additional 48 patients would be required to maintain the power at 80% for B, or approximately a 15% increase in the required sample size.

**Table 6.** Observed rates of non-fatal myocardial infarction or death (study by Poldermans et al.).

		Treatment B		
		Present	Absent	Total
Bisoprolol	Present	0.044	0.100	0.072
	Absent	0.150	0.300	0.225
	Total	0.097	0.200	

## Conclusions

We have shown how the power for one treatment in a binary outcome 2 × 2 factorial trial can change depending on the observed effect of the other treatment. Numerical evaluations illustrated how power is affected, primarily by the control event rate and the sample size, and by the measurement scale on which interactions are defined. Table 7 provides a condensed summary of the findings. Note that what is considered “larger” or “smaller” control event rates depend strictly on the other parameters, but as a guideline, this distinction occurs at control event rates of approximately 0.6.

**Table 7.** Summary of main findings.

		Estimate for first treatment	Effect on power for second
Scale	Control event rate	greater than postulated value?	treatment
Multiplicative	Smaller	Yes	Decrease
	Smaller	No	Increase
	Larger	Yes	Decrease
	Larger	No	Increase
Odds ratio	Smaller	Yes	Decrease
	Smaller	No	Increase
	Larger	Yes	May Increase or Decrease
	Larger	No	May increase or decrease
Additive	Smaller	Yes	Increase
	Smaller	No	Decrease
	Larger	Yes	Decrease
	Larger	No	Increase

When the treatments do not interact on the additive scale, the power relationship may either increase or decrease depending on the control event rate. As the control event rate approaches 1, observed effects for the first treatment that are greater than its assumed value lead to power decreases for the second treatment, and *vice versa*. However, when the control event rate approaches smaller values, the opposite pattern is observed. Additive effect estimates for the first treatment that are greater than its assumed value now result in power increases for the second treatment, and *vice versa*. Where this change in behaviour occurs is generally between control event rates of 0.5 and 0.6. Across a large range of parameter combinations, the change in power (either increase or decrease) tends to be less than 5% if the additive effect of the first treatment is within 0.05 of its assumed value. In extreme cases where the observed effect of the first treatment is much greater than the assumed value (e.g., a difference of 0.2), power changes of 10%–15% are possible, but these are less likely to occur in practical studies.

When treatments do not interact multiplicatively, an observed effect of one treatment that is greater than its originally postulated value always leads to a power decrease for the other treatment. In practice, this loss in power is typically between 1% and 3% for each decrement of 0.05 the first treatment's observed multiplicative effect is from its postulated value. Conversely, if the first treatment effect is smaller than expected initially, a 1%–3% increase in power is observed for each 0.05 increment in the postulated multiplicative effect over its assumed value. At high control event rates, large losses in power (e.g., 40%) are theoretically possible, but these only occur in extreme cases where the observed effect of the first treatment is much greater than expected (e.g., an observed effect of 0.5 versus a postulated effect of 0.05). We also found that the power decrease is greatest for an intermediate value of the second treatment's postulated effect, with the specific value depending on the other parameters.

Finally, when the treatments do not interact on the odds ratio scale, the power relationship is similar to that seen in the multiplicative case, as long as the control event rate is small. This is as expected because odds ratios and risk ratios are numerically similar in this case. However, at higher control event rates, the odds ratio power curves show some concavity. Larger observed effects for the first treatment lead to small power increases for the second treatment, eventually changing to power decreases if the observed effect of the first treatment takes extreme values. In the cases we considered, the change in power with the odds ratio scale tends to be small to moderate (no more than 15%), even for extreme first treatment effects, regardless of sample size or control event rate. As before, the greatest change in power tends to be for an intermediate value of the postulated effect of the second treatment and varies based on the other parameters.

In examining our results across different scales of measurement and parameter settings, we found that the observed magnitude of one treatment effect can have a noticeable impact on the power for the other treatment. Although large changes in power for the second treatment are possible, only small to moderate changes can be anticipated if the first treatment effect is reasonably close to its postulated value. In practice, observed treatment effects that are larger than their planned value are not common,^12^ and when they do occur, stopping rules for the benefit may be invoked. A consideration in the decision to stop a trial early for benefit, is that an interim analysis will then give an over-estimate of the true treatment effect, particularly with small sample sizes.^13–15^

As seen in a number of our results, even a moderate change in power for treatment may still represent a considerable change in the relative type II error rate (e.g., a 10% drop in power from 0.8 to 0.7 represents a 50% relative increase in the type II error rate, from 0.2 to 0.3). Our results also suggest that it may not be ideal to analyse both treatments simultaneously, even in situations where that is possible. Analyzing the effect of one treatment first, while still blinded to the effect of the second, affords investigators the opportunity to adjust the sample size prior to the second treatment analysis if a decrease in power has occurred. In other words, investigators gain the ability to adapt the study to prevent a loss of power.

Additional considerations relate to which treatment effect should be analysed first. If the postulated effects for the two treatments differ markedly, it may be preferable to first analyse the one with the larger anticipated effect. The justification for this stems from the fact that the planned total sample size for the trial is usually calculated as the larger of the two treatment-specific sample sizes. This approach yields power at the required level for the smaller effect and excess power for the larger effect. Hence, the power for the treatment with the smaller effect is at greater risk of falling below its desired power level, depending on what is observed for the other treatment. Therefore, analysing the treatment with the larger postulated effect first permits a potential adjustment in sample size in order to maintain power for the other treatment.

In situations where the postulated effects of both treatments are the same, it may instead be preferable to first analyse the treatment for which the investigators are less confident in their assumptions. For example, this may pertain in a trial where a new treatment is being compared to an established (or standard) treatment whose effect has been extensively studied previously. One might reasonably assume that the treatment with the less well-established effect is more likely to yield an effect that is materially different from its postulated value, so investigators should be concerned that this less predictable effect may importantly influence the power of the other treatment. By analyzing the new treatment first, investigators will be better positioned to adapt the required sample size for the second treatment if the first analysis suggests it now has decreased power. Alternatively, if the first analysis indicates increased power, no action is required. In either case, analyzing the treatment with the less predictable effect is beneficial for the second treatment analysis.

**Figure 6.**
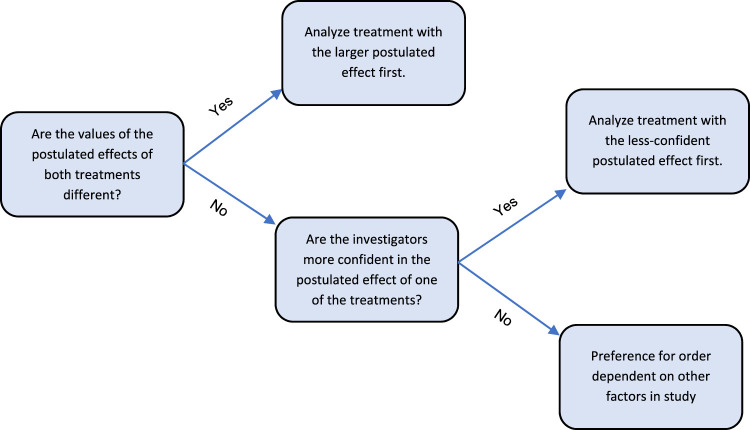
Recommendations for order of treatment analyses.

Figure 6 summarizes our recommendations on which treatment to analyze first. In practice, the preferred sequence of analyses may depend on other factors in each specific trial.

This investigation is limited in several ways. First, we examined only factorial trials with two treatments and a binary outcome. The issues discussed here in factorial trials with continuous outcomes remain open to future investigation. Use of continuous outcomes would introduce additional concerns, such as potentially heterogeneous variances or non-normal effects in the outcome; and these factors may be relevant to the issue of how to sequentially analyse the treatments.

Second, we exclusively considered scenarios where the entire sample is available to estimate the effect of the first treatment, so we did not consider cases where this estimate comes from an interim analysis on a subset of patients, which could nevertheless still affect the marginal estimates for the second treatment. For example, If an interim analysis for the first treatment leads to randomization for that treatment (or control) being stopped early for apparent benefit or harm, the trial can nevertheless continue to randomize patients to the second treatment. There will still be an impact on the marginal event rates for the second treatment, which will derive from a weighted average of the interim factorial data and the subsequent patients randomized to one treatment only. Furthermore, there will be some instability in the interim analysis because of smaller sample sizes being available at that point. That scenario remains an avenue for future research.

Third, we assumed no interaction between the treatments on a given scale of measurement. Ideally, factorial trials should be planned where this is actually true, particularly given that tests for the interaction are often weak. However, a more general investigation of the impact of interactions on the power and sample size questions that we have raised would be of interest. Finally, an additional extension of our work would be to cover the situation where different target power levels are specified for the two treatments in the trial.

Finally, note that we only considered scenarios where the sample sizes were equal in each of the four treatment combinations of a factorial trial. Further work would be required to examine the case of unequal sample sizes.

In conclusion, our results provide a framework for investigators to understand how the power of one treatment in factorial trials is influenced by the effect of the other treatment. In particular, they indicate how a careful analysis of the first treatment can lead to suitable modification in the study design, thus avoiding a potential loss of power for the second treatment. In this regard, investigators should use particular vigilance in cases where the first treatment effect estimate differs greatly from its proposed value.
